# Audiological findings of a patient with H syndrome: case report

**DOI:** 10.1186/s43163-021-00185-8

**Published:** 2021-12-04

**Authors:** Diala Hussein, Büşra Altın, Münir Demir Bajin

**Affiliations:** 1 Department of Audiology, Hacettepe University Faculty of Health Sciences, Ankara, Turkey; 2 Department of Otolaryngology Head and Neck Surgery, Hacettepe University, Ankara, Turkey

**Keywords:** H syndrome, Sensorineural hearing loss, Genetic hearing loss, Case report

## Abstract

**Background:**

H syndrome is an autosomal recessive disorder caused by mutations in SLC29A3. Hyperpigmentation, hypertrichosis, hyperglycemia, and hearing loss are some characteristics of this disorder, and it has a prevalence of < 1/1000. The aim of this report is to spread awareness among otologists, audiologists, and pediatricians about this syndrome and its audiological features.

**Case presentation:**

An 8-year-old male with a diagnosed H syndrome registered to our clinic with a complaint of hearing loss. The patient was diagnosed with hearing loss in a different clinic using only the air-conducted click auditory brainstem response test which showed wave V at 60 dB nHL for the right ear and at 80 dB nHL for the left ear. The initially performed pure tone audiometry (PTA) test in our clinic revealed a bilateral asymmetric hearing loss with a moderate sensorineural hearing loss in the right ear and a profound mixed hearing loss in the left ear. The performed air conducted click auditory brainstem response (ABR) result showed wave V at 55 dB nHL for the right ear and at 70 dB nHL for the left ear. Then, the repeated PTA test revealed a mild-severe sensorineural sloping hearing loss in the right ear and a severe sensorineural hearing loss in the left ear.

**Conclusion:**

Although hearing thresholds in H syndrome could be within normal limits in some patients, sensorineural hearing loss is an important characteristic feature for this syndrome. Sensorineural hearing loss could be progressive or of sudden onset and ranges from mild to profound. Thus, it must be taken into consideration to apply the audiological follow-up regularly and paying attention to the patient’s complaints; also, a regular follow-up for language development of children with H syndrome and for the hearing aids is advised.

## Background

H syndrome is an autosomal recessive disorder, which its name came from the major clinical and laboratory findings of cutaneous hyperpigmentation and hypertrichosis, hepatosplenomegaly, heart anomalies, hearing loss, hypogonadism, low height, and hyperglycemia [9, 10]. H syndrome is caused by a mutation in the SLC29A3 gene that encodes the human equilibrative nucleoside transporter hENT3 [9, 10].

Here, we describe the audiological findings of a Syrian boy who was diagnosed with H syndrome.

## Case presentation

**Fig. 1:**
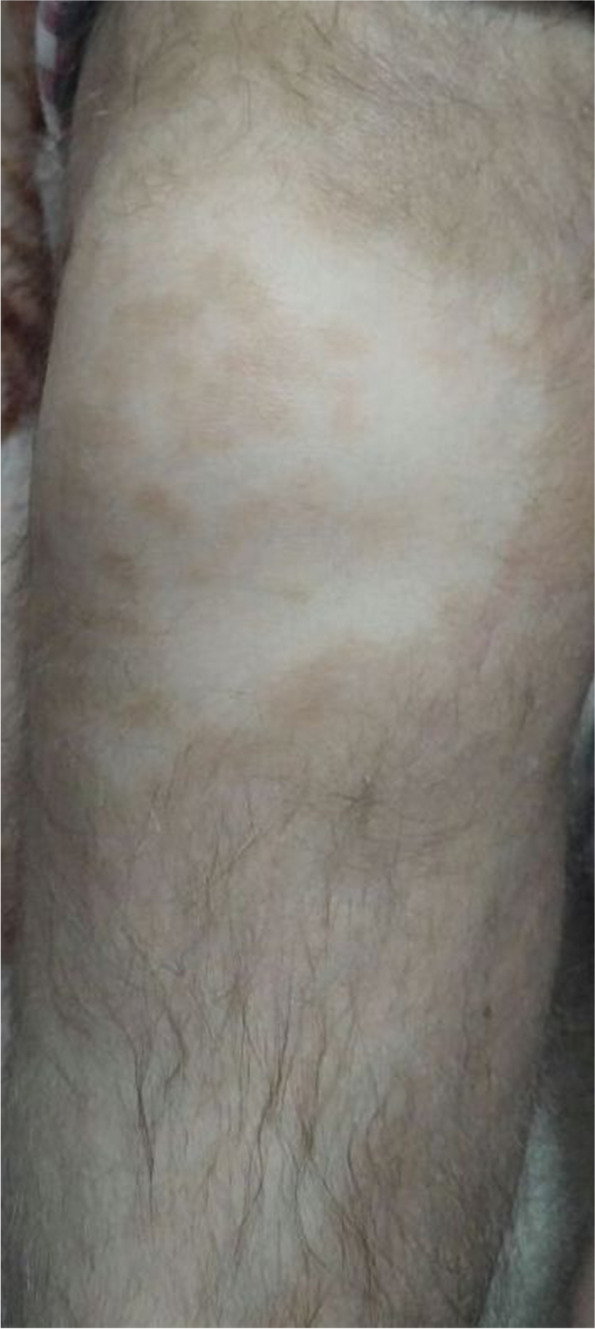
The child’s leg showing hyperpigmentation and hypertrichosis

Our patient is 8 years old first child of first-cousin consanguinity Syrian healthy parents. He has two smaller siblings without any complaint. According to the reports, a homozygous pathogenic variant in exon 6 of the SLC29A3 gene was detected. The child has heart anomalies (aortic stenosis), hyperpigmentation and hypertrichosis (Fig. 1), type 1 diabetes mellitus, hepatosplenomegaly, hard and fixed submandibular, and submental lymph nodes. Otherwise, he has a normal central nervous system, normal chest on evaluation, and normal male genitalia.

**Fig. 2:**
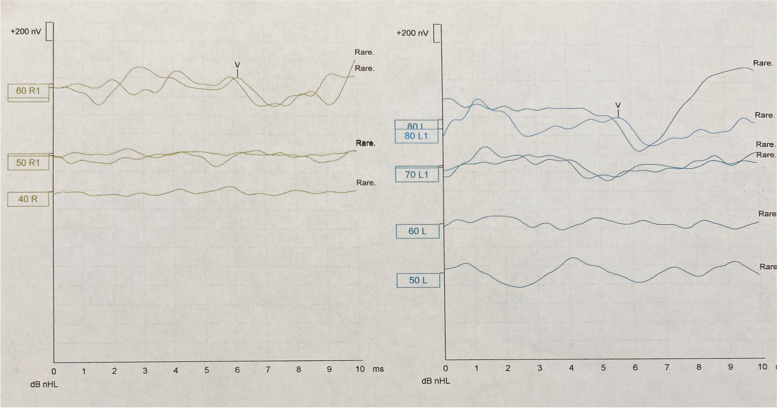
Result of the ABR which was done in a different hospital

At age of 3, the child started complaining about his hearing by saying that he is not hearing well and there is a strange sound in his ears. Getting older, he complained about ringing in his ears that wake him up sometimes. Also, his mother noticed his complaint about dizziness and imbalance which disappear after eating something. Unfortunately, through this period no audiological evaluation was done due to the available services and financial issues in the country where he was living. On September 23, 2019, at age of 8 years, the air-conducted click ABR test which was performed in a different clinic showed wave V at 60 dB nHL for the right ear and at 80 dB nHL for the left ear (Fig. 2).

**Fig. 3:**
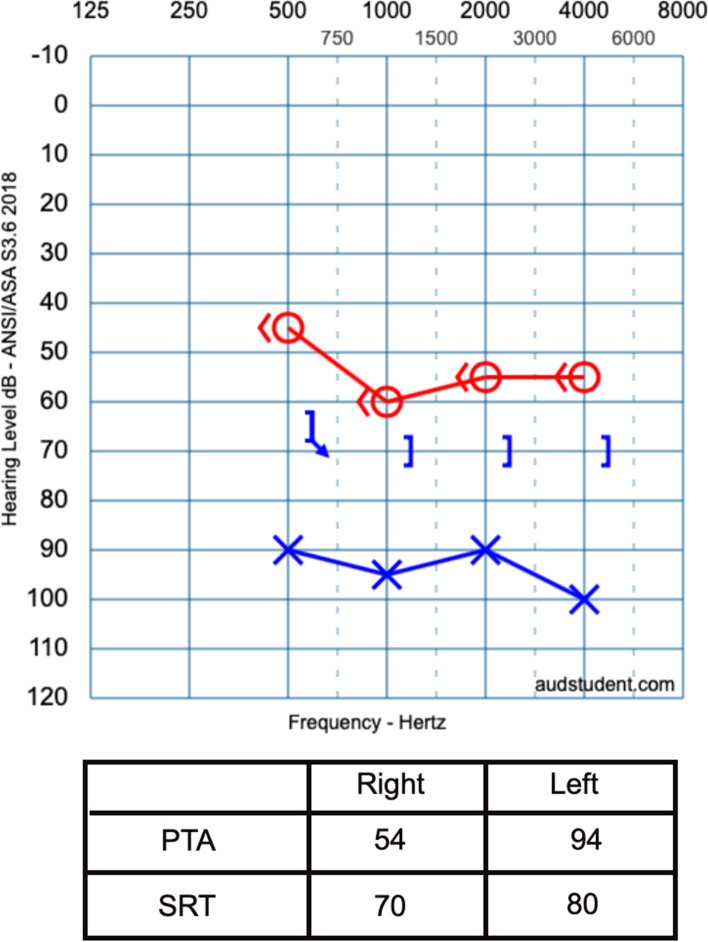
The result of the initial pure tone audiometry that performed at our clinic on November 29, 2019

On November 29, 2019, the child was referred to our clinic for audiological evaluation where tympanometry, acoustic stapedial reflex (ASR), transient evoked otoacoustic emissions (TEOAEs), and PTA tests were performed on the same day (Tables 1 and 2, and Fig. 3). The performed PTA with insert earphones showed a moderate sensorineural hearing loss in the right ear and a profound mixed hearing loss in the left ear. As the tympanometry results were normal, the child was referred to radiological assessment to investigate the reason for the air-bone gap in the left ear; also, an ABR appointment was planned.

**Table 1:** Results of the tympanometry and TEOAEs, November 29, 2019

	Right	Left
Tympanometry	Type A	Type A
TOAEs	Absent	Absent

**Table 2:** Results of the ipsilateral acoustic reflex, November 29, 2019

	500 Hz	1000 Hz	2000 Hz	4000 Hz
Right	100 dB	100 dB	100 dB	100 dB
Left	Absent	Absent	Absent	Absent

**Fig. 4:**
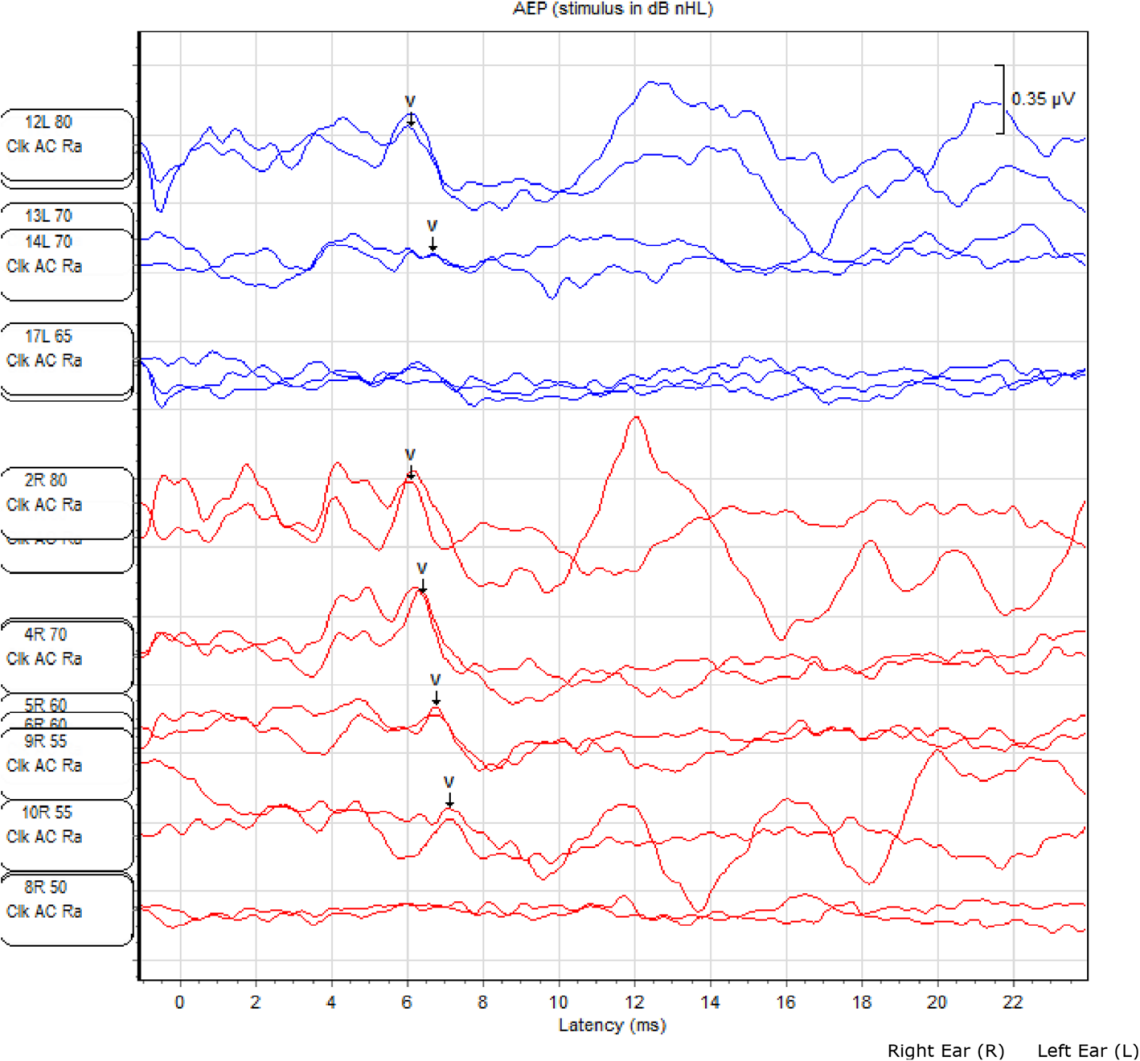
The result of the ABR performed at our clinic on January 10, 2020

**Fig. 5:**
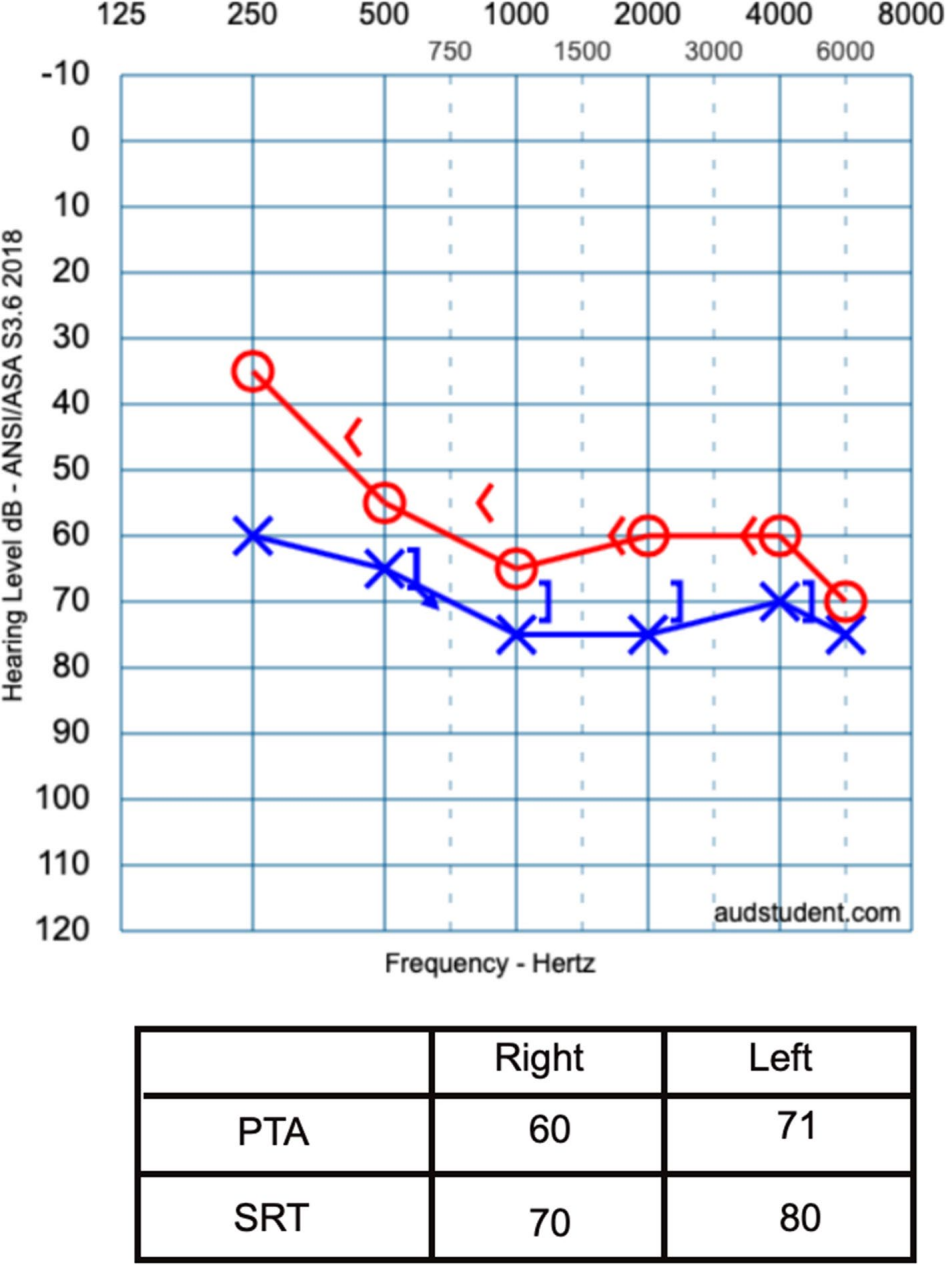
The result of the repeated pure tone audiometry performed at our clinic on February 10, 2020

On January 10, 2020, the air conducted click ABR test was performed in our clinic and showed wave V at 55 dB nHL for the right ear and at 70 dB nHL for the left ear (Fig. 4). The difference in the results of PTA and ABR tests led us to plan an appointment for further audiometric evaluation to assure the results. The PTA test that performed on February 10, 2020, revealed a mild-severe sensorineural sloping hearing loss in the right ear and severe sensorineural hearing loss in the left ear (Fig. 5).

On February 10, 2020, oculomotor assessment and balance tests also were performed. The patient was asked to follow the clinician’s finger with his eyes without head movement, and the assessments of gaze holding, pursuit, saccades, and vergence were performed. Oculomotor test results were normal and no nystagmus was revealed. Romberg’s test was negative. No refixation saccades were revealed with head movements to the right and left sides on head impulse test. The patient was asked to stand on a firm surface with the eyes open, and then closed. Postural sway was observed and could not stand with eyes closed. Lastly, the patient’s Gait was slow with a widened base.

The results of temporal bone magnetic resonance imaging and high resolution computed tomography revealed a bilateral normal temporal bone.

Hearing aids usage was recommended to improve the child’s hearing thresholds. The child was referred by us to a hearing aid center for earmold impression; then, hearing aid trying was done by another audiologist in our clinic, but unfortunately as his family could not afford the hearing aids’ price, they had registered in non-governmental organizations to help, but starting of COVID-19 pandemic delayed the process.

Necessary permissions were taken from the parents, and the informed consent form was signed by the father of the child.

## Discussion

A mutation in the SLC29A3 gene that encodes the human equilibrative nucleoside transporter hENT3 is the cause of the H syndrome [9, 10]. Sensorineural hearing loss is a distinctive feature of the H syndrome [7, 12]. It can be congenital, has a sudden onset, or be slowly progressive [9, 10]. Indeed, the degree of hearing loss in H syndrome patients ranges from mild to profound hearing loss. Nevertheless, some patients have completely normal hearing [1, 5, 14]. Also, hearing deterioration is extremely severe, resulting in bilateral profound deafness within few years [7].

Molho-Pessach et al. [11] investigated the clinical and molecular findings of the first 79 patients diagnosed with H syndrome. They found that sensorineural hearing loss is the third most common clinical finding as it is present in 53% of patients, with an average of 5.9 years as the age of onset for 21 patients with available data [11].

Even though the H syndrome is rare as it has a prevalence of < 1/1000, in Molho-Pessach et al. [11], the majority of cases were of Arab origin (55 patients of 79) [11]. Also, in Molho-Pessach et al. [9, 10], individuals of Arab-Palestinian origin carried a 1% frequency of alleles carrying the 2 most common disease-causing mutations (G437R, G427S) [9, 10].

Mitochondrial cytopathies are several diseases caused by a disturbance in one or more of the mitochondrial metabolic pathways, either by an unclear mutation in the DNA or by maternally inherited mitochondrial genome mutation [13]. One of the mitochondrial cytopathy-associated features is progressive hearing loss without vestibular failure [2]. Thus, the mitochondrial defect is thought to be the reason for deafness in some H syndrome patients [3]. In our patient, no balance disorder was detected by the initial vestibular assessment we could perform. As the dizziness and imbalance complaints were getting released after eating something, it is thought to be caused by diabetes.

Indeed, hENT3 is recognized as playing a major role in transferring the nucleosides into the mitochondria, thereby helping in mitochondrial DNA synthesis/ repair [6]. Mitochondria are responsible for cellular energy production as an integral component of the oxidative phosphorylation pathway [4], while cochlear processing requires substantial energy consumption; thus, it is affected by inefficiencies in the energy-producing apparatus that mitochondrial mutations would cause, resulting in deafness [4].

For our patient, the existed difference between the initial and latterly repeated PTA test results for the left ear is thought to be due to the tiredness our patient felt during the test, as he had more than one appointment in different policlinics on the same day. The audiological evaluation for our patient revealed a mild-severe sensorineural sloping hearing loss in the right ear and severe sensorineural hearing loss in the left ear, with bilateral normal tympanometry and absent TEOAEs, and a preserved ASR in the right ear. The reason of preserved ipsilateral ASR in the right ear and its absent in the left ear is thought to be due to Metz recruitment, as hearing loss of cochlear origin results in ASR thresholds within normal limits for levels of hearing loss up to 60 dB HL and to 70 dB HL in some cases [8].

These results support the necessity of supporting the subjective hearing tests with objective tests such as ABR in children with additional problems like our patient and also the necessity of regular audiological follow-up due to the risk of progressive hearing loss.

We had several limitations with this case; the family is living 12 h far away from our hospital, which forced them to plan all the needed appointments for the same day. This problem limited us to repeat the tests within a short period. Furthermore, the detailed vestibular assessment with laboratory tests (VNG, VEMP, posturography, etc.) was planned to be done, but because of the COVID-19 pandemic, it was postponed. Unfortunately, the patient passed away in July 2020.

## Conclusions

Hearing loss characteristics in H syndrome patients vary between mild to severe sensorineural hearing loss, and it could be of progressive or sudden onset. Although the H syndrome is rare, it has a higher prevalence in some countries than others, especially in the Middle East countries. This report aimed to spread awareness about the audiological findings in H syndrome especially among otologists, audiologists, and pediatricians who work in the Middle East countries. Regular audiological and language development follow-up and using objective and subjective tests during audiological evaluation is highly recommended; also, a regular hearing aids checking is recommended as the hearing loss could be progressive.

## Data Availability

The datasets used and/or analyzed during the current study are available from the author on reasonable request.

## Abbreviations

PTA: Pure tone audiometry
ABR: Auditory brainstem response
TOAEs: Transient otoacoustic emissions
hENT3: Human equilibrative nucleoside transporter
PTA: Pure tone average
SRT: Speech recognition threshold
ASR: Acoustic stapedial reflex
VNG: Videonystagmography
VEMP: Vestibular evoked myogenic potential
